# Determination of Cyanotoxins and Phycotoxins in Seawater and Algae-Based Food Supplements Using Ionic Liquids and Liquid Chromatography with Time-Of-Flight Mass Spectrometry

**DOI:** 10.3390/toxins11100610

**Published:** 2019-10-22

**Authors:** Claudia Giménez-Campillo, Marta Pastor-Belda, Natalia Campillo, Natalia Arroyo-Manzanares, Manuel Hernández-Córdoba, Pilar Viñas

**Affiliations:** Department of Analytical Chemistry, Faculty of Chemistry, Regional Campus of International Excellence “Campus Mare Nostrum”, University of Murcia, E-30100 Murcia, Spain; claudia.gimenez@um.es (C.G.-C.); marta.pastor@um.es (M.P.-B.); ncampi@um.es (N.C.); natalia.arroyo@um.es (N.A.-M.); hcordoba@um.es (M.H.-C.)

**Keywords:** marine toxins, ionic liquids, time-of-flight mass spectrometry, seawater, food supplements

## Abstract

An analytical procedure is proposed for determining three cyanotoxins (microcystin RR, microcystin LR, and nodularin) and two phycotoxins (domoic and okadaic acids) in seawater and algae-based food supplements. The toxins were first isolated by a salting out liquid extraction procedure. Since the concentration expected in the samples was very low, a dispersive liquid–liquid microextraction procedure was included for preconcentration. The ionic liquid 1-hexyl-3-methylimidazolium hexafluorophosphate (80 mg) was used as green extractant solvent and acetonitrile as disperser solvent (0.5 mL) for a 10 mL sample volume at pH 1.5, following the principles of green analytical chemistry. Liquid chromatography with electrospray ionization and quadrupole time of flight-mass spectrometry (LC-Q-TOF-MS) was used. The selectivity of the detection system, based on accurate mass measurements, allowed the toxins to be unequivocally identified. Mass spectra for quadrupole time of flight-mass spectrometry (Q-TOF-MS) and Q-TOF-MS/MS were recorded in the positive ion mode and quantification was based on the protonated molecule. Retention times ranged between 6.2 and 18.3 min using a mobile phase composed by a mixture of methanol and formic acid (0.1%). None of the target toxins were detected in any of the seawater samples analyzed, above their corresponding detection limits. However, microcystin LR was detected in the blue green alga sample.

## 1. Introduction

Cyanotoxins and phycotoxins are secondary metabolites produced by harmful algal blooms of toxic phytoplankton species or photosynthetic microorganisms such as cyanobacteria (green–blue algae), which are found in both freshwater habitats and marine environments [1]. The toxins are bioaccumulated by marine organisms, meaning that they may enter the human food chain. In humans, they can cause acute symptoms as gastroenteritis, fever, irritation and liver damage. Their chronic effects include neurotoxicity and carcinogenesis [2]. Microcystins (MCs) and nodularins (NODs) are the most abundant cyanotoxins in surface waters and it is difficult to obtain safe drinking water when they are present. MCs may also be found in food supplements, and some countries have set a maximum permitted limit of 1 μg g^−1^ [3]. Phycotoxins are found in molluscs such as oysters, mussels, clams and, to a lesser extent, in fish, crabs, turtles and algae [4], and their presence can produce toxic episodes in human health. Domoic acid (DA) and okadaic acid (OA) are the phycotoxins studied in this work.

Based on their chemical properties, marine shellfish toxins can be divided in two different classes: hydrophilic and lipophilic. Toxins associated with amnesic and paralytic syndromes are hydrophilic and have a molecular weight of less than 500 Da (e.g., DA), while toxins responsible for neurological and diarrhetic poisoning are strongly lipophilic and have a molecular weight of more than 600 Da, (e.g., OA, MCs and NODs) [5].

The European Council Regulation No 853/2004 has established maximum levels for certain marine biotoxins in molluscs intended for human consumption [6]. The maximum for the sum of OA, dinophysistoxins (DTXs) and pectenotoxins (PTXs) has been established at 160 μg kg^−1^ in edible shellfish. The World Health Organization (WHO) recommends a maximum concentration of 1 and 20 μg L^−1^ for drinking and recreational water, respectively [7].

Biological, biochemical and chemical methods have been used to determine cyanotoxins and phycotoxins in a variety of samples, including tap, sea, lake, surface, environmental and treatment plants water [8,9,10,11,12,13,14,15,16,17,18,19,20,21], fish [19,22,23,24], molluscs [25], algae [8,14], dolphin tissues [26], cells of a *Planktothrix rubescens* strain isolated from a lake [27] and cyanobacterial blooms [28]. In general, physicochemical methods are preferred for the analysis of these toxins, and, in recent years, liquid chromatography coupled with different detectors has become the technique of choice. Diode array detectors (DAD) [8,9,16,17,18,27] and ultraviolet detectors [14,15] are the most commonly used. Currently, mass spectrometry, using different analyzers [8,10,12,19,22,23,25,26,29] is becoming popular for this type of toxins due to the selectivity and sensitivity it offers. Gas chromatography has also been used coupled to mass spectrometry for the analysis of total microcystins [24].

The low concentrations of toxins expected and the complexity of the samples make sample treatment the most difficult step in the whole analytical process. A clean-up and/or a preconcentration step is required to remove interferences and preconcentrate the toxins. Solid phase extraction (SPE) has been the conventional methodology in this respect, particularly in the case of water [8,9,10,11,12,14,17,18,19,29], algae [14], fish tissues [19] and cyanobacterial blooms [28]. Liquid–liquid extraction (LLE) has been applied for the treatment of cells of a *Planktothrix rubescens* strain isolated from a lake [27] and fish [23] and ultrasound assisted extraction (UAE) for tissue of clam *Macoma balthica* and algal samples [8]. In recent years, microextraction techniques have become more relevant because they comply with the principles of green analytical chemistry. Thus, solid phase microextraction (SPME) has been used for water samples [10,15] and fish tissues [24] and magnetic solid phase extraction (MSPE) for water [16,21,30] and fish tissues [20,22].

Dispersive liquid–liquid microextraction (DLLME) is a simple miniaturized technique based on a ternary solvent mixture of an extractant solvent, a disperser solvent and an aqueous phase. The high surface contact area offered by the microdrops of the extract solvent leads to high preconcentration factors. Ionic liquids (IL) are salts formed by a voluminous organic cation and a small anion; they have a high degree of thermal stability, very low vapor pressure, variable viscosity and miscibility with both organic and aqueous solvents, which makes them environmentally friendly compounds. Thus, instead of using toxic organic solvents, ILs can be used as extractants for DLLME, reducing the amount of solvent and the extraction time. Only two procedures based on ILs using DLLME [31] and cloud point extraction (CPE) [32] have been described in the literature for the determination of marine toxins in water samples.

Therefore, the aim of this work was to develop and validate a reliable and sensitive analytical procedure for the determination of three cyanotoxins, MC-LR, MC-RR and NOD, and two phycotoxins, DA and OA, in seawater samples and algae-based food supplements using an ionic liquid acting as extractant solvent for DLLME and liquid chromatography with electrospray ionization and quadrupole time of flight-mass spectrometry (LC-Q-TOF-MS) for the final separation and measurement steps. This is the first time that IL-DLLME has been used for the preconcentration of marine toxins found in food supplements.

## 2. Results and Discussion

### 2.1. Optimization of LC-Q-TOF-MS Conditions

Several reversed-phase C_18_ columns such as ACE Excel 3 C18-PFP (15 × 0.46 cm, 5 μm), Tracer Extrasil ODS2 (15 × 0.4 cm, 5 μm) and Zorbax Eclipse XDB-C18 (5 × 0.46 mm, 1.8 μm) were tested, leading to broadened peaks. The best results were obtained with a Zorbax RRHD Eclipse Plus C_18_ column (1.8 μm, 2.1 × 100 mm) with a 0.3 µm in-line filter. This column was also chosen by other authors for the analysis of different type of toxins [33]. Separation conditions were optimized using mixtures of methanol (MeOH) or acetonitrile and water in different proportions and at different pH values obtained with 0.1% *v*/*v* formic acid, 0.01 M acetic acid/acetate or 0.01 M phosphoric acid/phosphate buffer solutions. Narrower peaks for all compounds and better resolution were obtained working in an acidic medium, which was achieved using 0.1% *v*/*v* formic acid. Thus, the mobile phase was composed of a H_2_O:MeOH (95:5, *v*/*v*) mixture containing 0.1% formic acid (solvent A) and a H_2_O:MeOH (5:95, *v*/*v*) mixture containing 0.1% formic acid (solvent B). Using these solvent mixtures, both isocratic and gradient elution modes were tested. The isocratic elution was discarded because none of the tested proportions provided good resolution for the compounds, since DA, a strongly hydrophilic toxin, eluted closely to the dead time, while the rest of the toxins, all of a lipophilic character, were retained. Therefore, the gradient elution mode was chosen. For good resolution of all the compounds, different gradients were tested. The final elution program was as follows: 100% solvent A for 0.5 min, linear gradient from 0 to 99% solvent B for 20 min, isocratic step at 99% solvent B for 1 min, and finally return to the initial conditions (0% solvent B) in 3 min (total run time 24 min) at a flow rate of 0.4 mL min^−1^.

The exact mass measurements of each peak from total ion chromatograms (TICs) were obtained by quadrupole time of flight-mass spectrometry (Q-TOF-MS) detection in electrospray ionization (ESI) positive mode for the protonated molecule, and used for quantification purposes and confirmation. The analyses were carried out using the extracted ion chromatogram (EIC) obtained for the protonated molecule of each analyte with a 20 ppm window. The analytical signals used for quantification were the peak areas of the EICs.

Table 1 shows the retention times of the studied compounds, the experimental and theoretical *m/z*, instrumental error and the selected ions for each compound. The error (ppm) for the experimental *m/z* values with respect to the theoretical ones was calculated in percentage terms as the difference between the experimental value and the theoretical value divided by the theoretical mass and multiplied by 10^6^.

### 2.2. Optimization of the Microextraction Procedure

The experimental variables for the DLLME procedure (nature of extractant and disperser solvents, their volumes and pH and saline content of the aqueous phase) were optimized using a seawater sample fortified at 10 ng mL^−1^ with the cyano- and phyco-toxins in acidic medium.

Solvents denser than water (CCl_4_ and CHCl_3_) or with a density lower than that of water (1-undecanol and 1-octanol) were tested as extractant solvent, using 600 μL of each solvent, 1 mL of acetonitrile (ACN) as disperser solvent and 5 mL of seawater sample at pH 1.5. These solvents were found to be useful for extracting the cyanotoxins MCs and NOD, chloroform showing the best extraction efficiency. The chloroform volume was then optimized using volumes in the 500–800 µL range. The highest responses were achieved using 500 µL of CHCl_3_, which was selected, while higher volumes led to lower extraction efficiencies due to a dilution effect.

Marine toxins are strongly influenced by pH because they can exist in the form of different chemical species. For this reason, the optimal pH value for the aqueous phase was studied in a series of experiments at pH 1.5, 2.8 and 5 (using hydrochloric or phosphoric acid). At pH 5, none of the compounds were recovered from a seawater sample fortified at 10 ng mL^−1^, while the highest extraction efficiency was observed at a strongly acidic medium (pH 1.5). However, even when using the best experimental conditions, only the cyanotoxins were microextracted, while the phycotoxins DA and OA could not be extracted.

**Figure 1:**
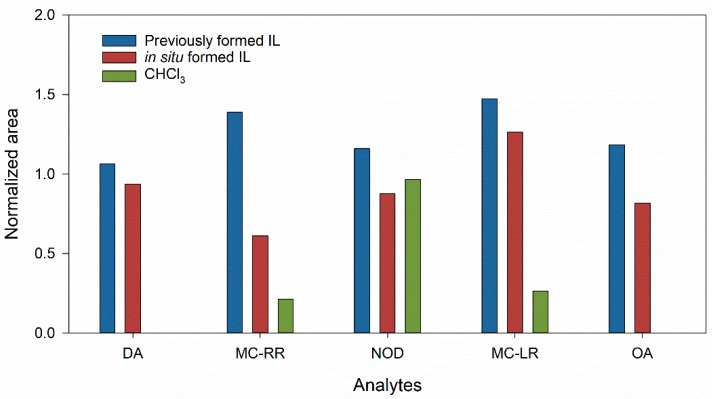
Comparison of the different extractant solvents used for the analysis of cyano- and phyco-toxins.

To improve the extraction of phycotoxins, alternative extractant solvents were assayed, including ILs. The use of ILs, as green extractant solvents, was evaluated using the previously formed IL, 1-hexyl-3-methylimidazolium hexafluorophosphate ([HMIM][PF6]), or the IL formed in situ by mixing lithium bis(trifluoromethylsulfonyl)imide ([LiTFSI], cation component) and 1-hexyl-3-methylimidazolium chloride ([HMIM Cl], anion component). When the DLLME procedure was carried out using the previously formed [HMIM][PF6], the addition of a disperser solvent was necessary, so 100 mg of the IL with 500 µL of ACN were rapidly injected into the aqueous phase at pH 1.5. On the other hand, in the DLLME procedure using the in situ formed IL, the disperser solvent was not necessary due to the metathesis reaction between both cation and anion components, which originated nanodroplets of the water-insoluble [LiTFSI][HMIM Cl] in the sample medium. In this case, 300 µL of a 1 M aqueous solution of each component (first the positive and then the negative) were added to the aqueous phase. Both assayed ILs preconcentrated the five marine toxins (cyano- and phycotoxins), the extraction efficiency for cyanotoxins being even better than that achieved using chloroform (Figure 1). A comparison of the extraction efficiencies using the previously formed and the in situ formed IL is shown in Figure 1. As can be seen, higher signals were generated when the previously formed [HMIM][PF6] was used.

The variables influencing the IL-DLLME procedure (amount of extractant solvent, volume of disperser solvent and sodium chloride percentage in the aqueous phase) were closely related, and a central composite design (2^3^ + star, face centered), with three spaced central points, involving 17 runs, was used as an approach to generate the response surface, using the area as analytical response. Ultrapure water fortified with the marine toxins at 10 ng mL^−1^ was used for this purpose. The above mentioned factors were studied in the following ranges: ACN volume (250–1000 μL); amount of [HMIM][PF6] (80–100 mg); and sodium chloride percentage (0–5%). Temperature, extraction time and stirring speed were not considered as variables because equilibrium was reached quickly. The centrifugation speed was fixed at 3000 rpm for 3 min.

**Figure 2:**
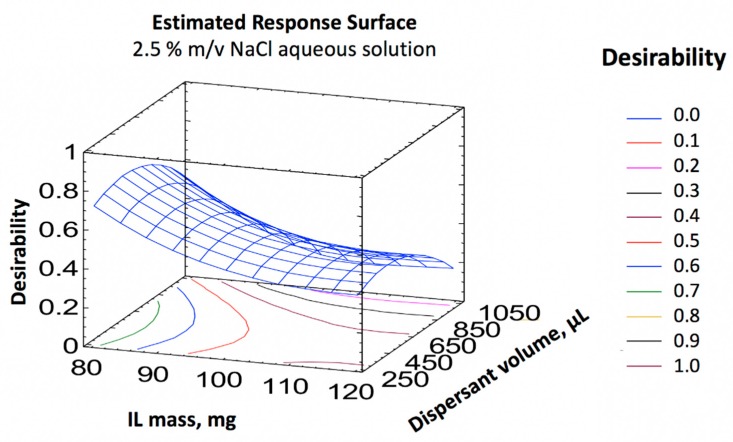
Response surface graphs obtained using the desirability function, considering only the responses of OA and DA.

The volume of disperser solvent and amount of IL had a significant effect on the DA and OA signals, but none of the studied variables had a significant effect in the case of the other toxins. As a result, multiple response optimization by using the desirability function was carried out considering only the responses for DA and OA, since the variables only had a significant effect on these toxins (Figure 2). The determination coefficients (*R*^2^) were 85.0% and 80.3% for DA and OA, respectively, confirming the suitability of the design. The optimal conditions for maximum extraction efficiency using the IL-DLLME procedure was 80 mg of [HMIM][PF6], 500 µL of ACN and 2.5% *m*/*v* NaCl in the aqueous phase.

The optimal amount of the sample was then optimized to achieve higher sensitivity. When 5 and 10 mL of seawater were evaluated higher signals were obtained for the 10 mL volume, which was selected. When the mass of the food supplement was varied between 0.1 and 1 g, sensitivity increased up to 0.3 g, while higher amounts of sample led to dirty extracts, which were unsuitable to be directly injected into the LC, so a 0.3 g sample mass was selected.

### 2.3. Method Validation

International guidelines [34] were followed to validate the proposed methodology. Linearity, sensitivity, precision and trueness were evaluated using calibration graphs, detection and quantification limits, repeatability and recovery studies, respectively.

Standard calibration graphs were obtained and, in addition, matrix-matched calibrations were obtained using the standard addition method on the seawater samples and food supplement samples and applying the IL-DLLME procedure.

All graphs were obtained using six concentration levels ranging from 5 to 30 ng mL^−1^ in duplicate experiments. The slopes of the calibration graphs obtained using IL-DLLME with standards and those obtained by standard additions to the seawater and food supplement samples, showed significant differences at 95% confidence level, as confirmed by ANOVA tests, the *p*-values obtained being lower than 0.05 for all compounds. Because the samples showed different suppression signals (matrix effect), the quantification for each sample had to be carried out using a model matrix (matrix-matched method). Linearity of the IL-DLLME procedure was obtained for the whole range studied (5–30 ng mL^−1^ and 50–1000 ng g^−1^ for the seawater and food supplements, respectively, with an R^2^ above 0.99 in all cases). The sensitivity of the procedure was evaluated by calculating the limits of detection (LDs) and quantification (LQs) using the criteria of three or 10 times the signal to noise ratio, respectively. The results for the LDs are shown in Table 2, values were in the range 0.22–1.5 ng mL^−1^ for the toxins in seawater samples and 7–52 ng g^−1^ in the food supplements.

The precision of the method was evaluated in terms of repeatability (intraday analysis) applying the IL-DLLME procedure to three aliquots of the same food supplement sample at 10 ng mL^−1^ level injected in triplicate (instrumental replicas) on the same day (*n* = 9). The results, expressed as relative standard deviations (RSD) of the peak areas, are shown in Table 2. As can be seen, RSD values were below 10% for all the analytes.

The trueness was evaluated by means of recovery experiments at two concentration levels (10 and 20 ng mL^−1^ for seawater and 300 and 900 ng g^−1^ for food supplements). Recoveries were calculated as measured concentration / actual (added) concentration × 100%, where measured concentration was determined using the obtained calibration curves. The results are shown in Table 3, and, as can be seen, recovery values ranged between 82% and 118% (*n* = 10).

**Figure 3:**
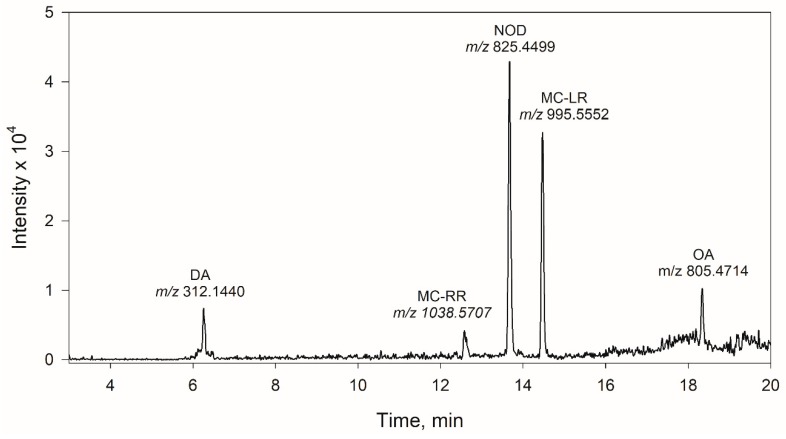
Extracted ion chromatogram obtained for marine phytoplankton (*Nannochoropsis gaditana*) fortified at 150 ng g^−1^ for DA, NOD and MC-LR, 300 ng g^−1^ for OA and MC-RR.

Figure 3 shows the extracted ion chromatogram obtained for a fortified sample of marine phytoplankton (*Nannochoropsis gaditana*) using IL-DLLME and LC-QTOF-MS. The chromatograms obtained from the non-fortified samples indicated the absence of interfering peaks at the retention times of the compounds.

### 2.4. Analysis of Seawater and Algae-Based Dietary Supplement Food Samples

Five seawater samples from south-eastern Spain were analyzed in duplicate experiments using the optimized IL-DLLME procedure prior to injection into the LC-Q-TOF-MS. Elution profiles demonstrated the absence of interfering compounds eluting at the retention times of the different toxins. The comparison of the retention times of the compounds in the standard mixture of standards and in the fortified samples, and especially the MS spectra, allowed identification of the toxins. None of the seawater samples contained any toxins above their LD.

Four samples of algae-based dietary supplements were analyzed in duplicate using the described methodology and only MC-LR was found in the blue-green algae supplement above its corresponding LD, but below its LQ, so it was not quantifiable. In the rest of the samples, if any analytes were present, it would have been at concentration levels below their LD.

## 3. Conclusions

Combination of the salting-out assisted liquid-liquid extraction (SALLE) extraction technique with a miniaturized procedure (DLLME with LC-TOF-MS) provided low detection limits for marine toxins, due to the high enrichment factors achieved and the high selectivity of the method. The method respects the principles of green analytical chemistry and can be defined as “green” due to the low consumption of toxic solvents. The developed procedure complies with the European standard requirements for analyzing traces of toxins based on microextraction techniques and represents a breakthrough in the miniaturization of the analytical laboratory. The method allows reliable analysis and is suitable for the determination of cyanotoxins and phycotoxins in seawater samples and algae-based food supplements using an ionic liquid (green solvent) as an extractant solvent.

## 4. Materials and Methods

### 4.1. Reagents

Chromatographic quality organic solvents, namely acetonitrile and methanol were obtained from Chem-Lab (Zedelgem, Belgium). Chloroform, carbon tetrachloride, 1-undecanol and 1-octanol were purchased from Sigma Aldrich (St. Louis, MO, USA). The ILs 1-hexyl-3-methylimidazolium hexafluorophosphate (C_10_H_19_F_6_N_2_P) [HMIM][PF6], lithium-imide bis(trifluoromethylsulfonyl)imide (LiC₂F₆NO₄S₂) [LiTFSI] and 1-hexyl-3-methylimidazolium chloride (C_10_H_19_ClN_2_) [HMIM Cl], were supplied by IOLITEC (Heilbronn, Germany). Other reagents, including formic acid and sodium chloride, were acquired from Sigma. Anhydrous magnesium sulfate from Análisis Vínicos (Tomelloso, Spain), hydrochloric acid from Riedel-de-Häen (Wunstarfer, Germany) and phosphoric acid from Panreac Química S.A (Barcelona, Spain) were also used. Water was previously purified using a Milli-Q system (Millipore, Bedford, MA, USA).

The cyanotoxin and phycotoxin standards were obtained from Cifga S.A. (Lugo, Spain). The standard solutions of cyanotoxins—microcystine LR (MC-LR) and RR (MC-RR) and nodularin (NOD)—were supplied at 10 µg g^−1^, while okadaic acid (OA) was supplied at 16 µg g^−1^ and domoic acid (DA) at 40 µg g^−1^. All were stored in the dark at −20 °C, and standard working solutions were prepared daily diluting the toxins with ultrapure water.

### 4.2. Instrumentation

The ultra-high performance liquid chromatography (UHPLC) system consisted of an Agilent 1290 Infinity II Series HPLC (Agilent Technologies, Santa Clara, CA, USA). The chromatographic system was a high-speed binary pump working with a flow rate of 0.4 mL min^−1^. The elution program was as reported in Section 2.1. The column was kept in a thermostatted compartment at 30 °C. The analytical column used was a ZORBAX RRHD Eclipse Plus C18 (1.8 μm, 2.1 × 100 mm) from Agilent Technologies (Diegem, Belgium) with an in-line filter of 0.3 µm (Agilent Technologies). Injection (20 μL) was carried out using an autosampler using 2 mL capacity vials supplied with 250 µL microinserts with a polymeric foot. Samples were kept at 5 °C.

The UHPLC system was coupled to an Agilent 6550 Q-TOF Mass Spectrometer (Agilent Technologies, Santa Clara, CA, USA) equipped with an ESI source (Agilent Jet Stream Dual electrospray, AJS-Dual ESI) operating in positive ionization mode and using the following operating parameters: capillary voltage, 4000 V; nebulizer gas pressure, 30 psi; drying gas flow, 16 L min^−1^; drying gas temperature, 130 °C; fragmentor voltage, 360 V; nozzle voltage, 500 V; 1 RF Vpp octapole, 750 V.

The MassHunter Workstation Data Acquisition software (Agilent Technologies, Rev.B.08.00, Santa Clara, CA, USA, 2017) was used to process the data. The exact mass measurements of each peak from the total ion chromatograms (TICs) were obtained by means of an automated calibration system that provides mass correction. The Q-TOF-MS spectrometer performed the internal mass calibration automatically, using a source of dual ionization with an automatic calibrant supply system, which introduced the flow from outside of the chromatograph together with small quantities (about 20 µL min^−1^) of a calibrating solution, reference mixture ES-TOF (Agilent), in ESI positive mode. Profile data in the range 50–1500 *m/z* were acquired for MS scans in 2 GHz extended dynamic range mode with 3 spectra, 333.3 ms/spectrum and 2675 transients/spectrum. Three collision energies (0, 10 and 40 V) were measured in each cycle.

The analyses were carried out using the extracted ion chromatogram (EIC) from the protonated molecule of each analyte, with a 20 ppm window. The exact mass of the protonated molecule was used for quantification purposes and confirmation. The analytical signals for quantification were the peak areas of the EICs.

An EBA 20 centrifuge (Hettich, Tuttlingen, Germany) and a Unicen-21 centrifuge (TQTech, Shenzhen, China) were used at 3000 rpm and 4500 rpm, respectively. An AS220/C/2 balance from Radwag Wagi Elektroniczne (Radom, Poland), an LLG-uniTEXER vortex agitator (LLG-Labware, Constantí, Tarragona) were used. An IKA-KS-130-Basic orbital agitator was used to treat the samples (IKA, Staufen, Germany), a rotavapor (BUCHI, Labortechnink:AG: Flawil, Switzerland) and an Xcelvap air-drying system (Horizon Technology, Salem, MA, USA) was also employed to evaporate the extracts. The polytetrafluoroethylene (PTFE) Uniprep Agilent filters (0.2 µm), nylon syringe filters (25 mm/0.22 µm) and 1 mL Nipro Syringe needle-free syringes were from Análisis Vínicos.

### 4.3. Samples

Sea water samples came from the “Mar Menor”, a lagoon located on the south-east coast of Spain. The method was optimized using a sea water sample free of the analytes. The analyzed water samples were taken from different areas and at different dates. A total of five samples were collected from Los Urrutias, Manzanares, and Castillitos between the third and seventeenth of September, 2018. The sample collection was always performed in the early morning at 30 cm from the surface, where the water column had a 1 m depth. Samples of 1 L were taken in polypropylene bottles and stored at 4 °C until analysis. Water temperature was in the range 26 to 29 °C and salinity varied from 42.0 to 43.2 g L^−1^.

Food supplement samples based on algae were also analyzed using the proposed methodology. Marine phytoplankton (*Nannochoropsis gaditana*), blue–green algae, spirulina (chlorophyll-rich blue-green algae) and spirulina commercialized as a food supplement from organic farming methods were obtained from local supermarkets.

### 4.4. Analytical Procedure

#### 4.4.1. Water Samples

The DLLME procedure involved rapidly injecting a mixture of 0.5 mL of ACN and 80 mg of 1-hexyl-3-methylimidazolium hexafluorophosphate (extractant solvent) into 10 mL of a sea water sample at pH 1.5 (attained using HCl). Then, a dispersion of micro-droplets of the extractant phase was formed and, after centrifugation for 3 min at 3000 rpm, the drop sedimented (volume 100 ± 10 µL) at the bottom of the conical tube and was collected with a micro syringe. The drop was diluted by adding 100 µL of MeOH before filtration through 0.2 µm Agilent Uniprep filters. The filtered extract was placed in a chromatographic glass microvial with an insert, and 20 µL were injected into the LC-TOF-MS.

#### 4.4.2. Dietary Algae-Based Food Supplement Samples

A solid-liquid extraction procedure was necessary prior to DLLME in order to extract the analytes from the solid matrix. For this, an aliquot of 0.3 g of the food supplement sample was weighed into a 15 mL centrifuge tube and 5 mL of water was added, subjecting the mixture to vortex agitation for 1 min. SALLE methodology was followed, adding 5 mL of ACN containing 5% *v*/*v* formic acid, 4 g of MgSO_4_ anhydrous and 1 g of NaCl. The mixture was shaken vigorously for 5 min in the orbital shaker and then centrifuged 5 min at 4500 rpm, thus obtaining a biphasic system. The organic phase (supernatant) containing toxins were recovered, evaporated with air at 40 °C and reconstituted with 500 µL MeOH, shaking in the vortex for 2 min. After that, 4.5 mL of water was added, and the extract was filtered with 0.2 µm nylon syringe filters. Then 5 mL of water containing 5% *m*/*v* NaCl was added, giving 10 mL of aqueous phase at 2.5% *m*/*v* NaCl to be used in the next stage. Subsequently, IL-DLLME extraction was carried out, using the procedure previously described for water samples.

## Figures and Tables

**Table 1:** Chromatographic and detection characteristics.

Compound	t_R_ (min)	*m/z* Theoretical	*m/z* Experimental	Error (ppm)	Q1, *m/z*	Q2, *m/z*
Domoic acid (DA)	6.22	312.1447	312.1440	−2.2	161.0963	266.1382
Microcystine-RR (MC-RR)	12.55	1038.5736	1038.5707	−2.8	135.0803	620.3387
Nodularin (NOD)	13.70	825.4511	825.4499	−1.5	135.0803	691.3774
Microcystine-LR (MC-LR)	14.46	995.5566	995.5552	−1.4	135.0803	861.4811
Okadaic acid (OA)	18.33	805.4738	805.4714	−3.0	769.4508	787.4621

**Table 2:** Analytical characteristic for the ionic liquid-dispersive liquid–liquid microextraction (IL-DLLME) method.

Compound	Seawater Limits of Detection (LD), ng mL^−1^	Food Supplements LD, ng g^−1^	Relative Standard Deviations (RSD), %
DA	0.39	13	8.2
MC-RR	1.4	52	10
NOD	0.22	7.5	9.5
MC-LR	0.33	11	7.7
OA	1.5	49	8.9

**Table 3:** Recovery studies.

Compound	Seawater, ng mL^−1^		Food Supplement, ng g^−1^	
	10	20	300	900
DA	94.3	99.2	84.4	105
MC-RR	118	102	87.4	103
NOD	94.8	99.3	81.7	102
MC-LR	108	101	113	99.1
OA	112	88.0	91.5	101
